# Predicting Cancer Tissue-of-Origin by a Machine Learning Method Using DNA Somatic Mutation Data

**DOI:** 10.3389/fgene.2020.00674

**Published:** 2020-07-14

**Authors:** Xiaojun Liu, Lianxing Li, Lihong Peng, Bo Wang, Jidong Lang, Qingqing Lu, Xizhe Zhang, Yi Sun, Geng Tian, Huajun Zhang, Liqian Zhou

**Affiliations:** ^1^School of Computer Science, Hunan University of Technology, Zhuzhou, China; ^2^Chifeng Municipal Hospital, Chifeng, China; ^3^Genesis Beijing Co., Ltd., Beijing, China; ^4^College of Mathematics and Computer Science, Zhejiang Normal University, Jinhua, China

**Keywords:** somatic mutation, machine learning, random forest, patients with carcinoma of unknown primary, tissue of origin

## Abstract

Patients with carcinoma of unknown primary (CUP) account for 3–5% of all cancer cases. A large number of metastatic cancers require further diagnosis to determine their tissue of origin. However, diagnosis of CUP and identification of its primary site are challenging. Previous studies have suggested that molecular profiling of tissue-specific genes could be useful in inferring the primary tissue of a tumor. The purpose of this study was to evaluate the performance somatic mutations detected in a tumor to identify the cancer tissue of origin. We downloaded the somatic mutation datasets from the International Cancer Genome Consortium project. The random forest algorithm was used to extract features, and a classifier was established based on the logistic regression. Specifically, the somatic mutations of 300 genes were extracted, which are significantly enriched in functions, such as cell-to-cell adhesion. In addition, the prediction accuracy on tissue-of-origin inference for 3,374 cancer samples across 13 cancer types reached 81% in a 10-fold cross-validation. Our method could be useful in the identification of cancer tissue of origin, as well as the diagnosis and treatment of cancers.

## Introduction

Researches have proved that hepatitis C virus (HCV) and hepatitis B virus (HBV) are the main causes of liver cancer, and liver cancer can be primary or metastatic, where metastatic liver cancer accounts for 5% (Hu and Ludgate, 2007; Lin et al., 2013). Studies have shown that Epstein–Barr virus (EBV) infection is one of the important causes of nasopharyngeal carcinoma (Hui et al., 1998; Krishna et al., 2006). Tsai et al. (1996) carried out numerous experiments and found that EBER1 expression is abundant in primary nasopharyngeal carcinoma, which may metastasize to lymph nodes. Numerous studies have shown that *Helicobacter pylori* (HP) is associated with gastric cancer (Farinati et al., 1993; Gonzaga et al., 2002; Geng and Zhang, 2017). Gastric cancer is one of the most common malignant diseases in the world, where metastasis often occurs, and there are histological differences between primary and metastatic gastric cancer (Wang et al., 2008). In most cases, viruses are a major cause of cancer. Metastatic cancer brings great adversity to the follow-up diagnosis and treatment. Some biomarkers are related with metastasis of cancer. Chen et al. (2016) carried out researches on the differential expressed proteins and found two biomarkers related with lung adenocarcinoma. Xiuping et al. (2016) found that NTN4 is associated with breast cancer cell migration and invasion via regulation of epithelial–mesenchymal transition–related biomarkers. Differentially expressed genes between metastatic tissue samples and nonmetastatic tissue samples can be molecular biomarkers for gastric cancer metastasis (Li et al., 2016).

In clinical diagnosis, metastatic cancer is a common phenomenon and a great challenge for determination of the primary site of a tumor. In all cases of cancer diagnoses, 3–5% of patients are confirmed as carcinoma of unknown primary (CUP) (Shaw et al., 2007). Cases of CUP are usually heterogeneous and can make diagnosis and treatment of pathological and clinical cases difficult (Rizwan and Zulfiqar, 2010). In the recent years, immunohistochemistry was a crucial method for classification of cancer and identify the primary site of a tumor and made great contributions to CUP identification (Huebner et al., 2007; Voigt, 2008; Centeno et al., 2010; Kandalaft and Gown, 2015; Janick et al., 2018). However, immunohistochemistry is labor-intensive and applicable to small-scale sample data, and it is difficult to overcome the bottleneck in classification accuracy.

Computed tomography (CT) and positron emission tomography are good medical imaging tools for identifying cancer tissue and predicting the primary site of a tumor (Fencl et al., 2007; Kwee et al., 2010; Fu et al., 2019). CT and PET identify tumors with an accuracy of 20–27% and 24–40%, respectively (Ambrosini et al., 2006). Obviously, the prediction performance is too poor to reach a satisfying degree. Moreover, medical images usually generate large-scale data, and limitations of image processing technology also bring about great difficulty in application. Identification of tissue origin utilizing medical imaging still remains conservative.

Recently, the use of molecular profiling has become a popular method to infer the primary site of a tumor. In addition, the combination of machine learning method and molecular profiling has been proven to be better than the utilization of immunohistochemistry for undifferentiated or poorly differentiated tumors (Oien and Dennis, 2012). Combination of methylation and copy number variation can contribute to cancer classification and tissue origin identification (Hoadley et al., 2014). Küsters-Vandevelde et al. (2017) suggested that metastatic behavior of a tumor is closely associated with specific copy number variations, as the methylation profile of meningeal melanocytic metastatic tumor was found to be similar as to that of the primary site. Although metastasis of cancer occurs, methylation and copy number variation are still in accordance with those of the primary origin. Particularly, gene expression data were frequently used in identification of the primary site of a tumor (Erlander et al., 2004; Qu et al., 2007; Gross-Goupil et al., 2012; Greco, 2013; Hainsworth et al., 2013). Erlander et al. (2011) proved that the value of gene expression detected in metastasis is the same as that detected in the primary origin when metastatic cancer occurs. Centeno et al. (2010) carried out numerous experiments with the proposed hybrid model, which utilized immunohistochemistry and gene expression profiling, and obtained classifier accuracies of 89, 88, and 75% for cross-validation datasets, independent test sets, and institutional independent test sets, respectively. Rosenwald et al. (2010) gained an accuracy of 85% on prediction of the primary site of cancer with the use of the KNN algorithm and micro-RNA quantitative reverse transcription–polymerase chain reaction test. Bloom et al. (2004) explored a method based on the artificial neural network with gene expression profiling to infer the tumor origin and thus aid in making a correct pathological diagnosis.

Somatic mutation data can also be utilized to identify tissue origin. Sheffield et al. (2016) revealed that mutation of the *IDH1* gene in patients with cholangiocarcinoma can be used to infer the primary site of the malignant tumor. Dietlein and Eschner (2014) and Lawrence et al. (2014) explored a method using mutation spectra to predict the primary site of cancer and obtained a specificity of 79%, showing that the enrichment of mutation in tumor-specific genes can be effective for primary tissue tracing. Relatively comprehensive research was conducted by Marquard et al. (2016), using somatic mutation data, base substitution frequency, trinucleotide base substitution frequency, and copy number aberrations. The best results with accuracy of 87.6% were obtained using a combination of copy number status, trinucleotide context base substitution frequencies, and somatic point mutations. However, it is complicated that each cancer was trained with a classifier. Moreover, the best performance was achieved using three molecular profiling, in which data collection is challenging.

Use of copy number variation, methylation, and gene expression to predict the primary site of a tumor has been a hot spot. However, research of predicting tissue origin using mutation data has made little progress. This current study proposed a new method using somatic mutation data to predict the primary site of cancer. The International Cancer Genome Consortium (ICGC), together with machine learning methods could improve the predictive performance. Here, the random forest algorithm (Sandri and Zuccolotto, 2006) was selected as a gene selection algorithm, and the logistic regression algorithm (Zhang et al., 2014; Pranoto et al., 2015) was utilized to establish a classifier. Performance evaluation was judged by metrics, such as accuracy and specificity. Functional annotation and enrichment of specific gene set were settled by R packages.

## Materials and Methods

### Data Preparation

We downloaded the somatic mutation data from ICGC database version 28^1^. The format of the gene name was Ensembl Gene ID. A total of 19,730 samples were obtained. We duplicated the samples according to chromosomal features, locus in chromosome, donor-id, and gene-affected. Sample data of 57 types of cancer were preliminarily extracted. Somatic mutation data cannot identify the primary site of some cancers. Samples with primary and metastasis of 13 types of common cancers were used to predict tissue origin (Table 1). Data were further filtered, and we generated an *S* × *G* matrix, where *S* represents the number of samples and *G* represents the number of genes included.

**TABLE 1:** Distribution of samples with 13 cancers.

Cancer Types		Samples	
	
Type	Abbreviation	Primary	Metastasis
Biliary tract cancer	BTCA	310	0
Chronic myeloid disorders	CMDI	136	0
Colorectal cancer	COCA	317	4
Gastric cancer	GACA	708	0
Brain lower-grade glioma	LGG	508	0
Liver cancer	LIRI	258	0
Soft tissue cancer	LMS	67	0
Malignant lymphoma	MALY	152	89
Skin cancer	MELA	183	0
Nasopharyngeal cancer	NACA	21	0
Pancreatic endocrine neoplasms	PAEN	87	2
Renal cancer	RECA	432	0
Skin adenocarcinoma	SKCA	52	48
Total		3,219	155

### Feature Selection

As mutation detection of tissue-specific gene is time consuming and costly, a balance between performance and number of genes used is necessary. Existing feature selection algorithms such as Lasso and Principal Component Analysis (PCA) (Malhi and Gao, 2005; Muthukrishnan and Rohini, 2016) have been largely used as a tool for feature processing. Here, we used the random forest algorithm (Breiman, 2001; Sandri and Zuccolotto, 2006) for feature selection. It can handle a large number of input features and assess their importance, and its learning process is fast. Tt is a type of ensemble learning algorithm and is composed of a CART (classification and regression tree). In each tree, $\sqrt{g}$ was used, where *g* denotes the gene number. The process of feature selection was explained by the splitting of nodes. The Gini index was used to determine which feature should be selected as most important and was calculated by the following Eq. 1:

(1) $$\text{Gini}\,\left(p\right)=\sum_{k=1}^{K}p_{k}\,\left(1-p_{k}\right)=1-\,\sum_{k=1}^{K}p_{k}^{2}$$

In a node, *p* denotes the weight represented as frequencies of cancers, *k* denotes the total cancer number, and the weight of *k*-th cancer is denoted by *p*_*k*_. We calculated feature importance scores of the *i*-th gene in a node, which was represented by a decrease in the Gini index value. This was calculated by Eq. 2:

(2) $$V\,I\,M_{i\,m}^{\left(G\,i\,n\,i\right)}=G\,I_{m}-G\,I_{l}-G\,I_{r}$$

*M* was used as the set of nodes. *m* denotes a node in *M*. Thereafter, we selected the *i*-th gene for splitting. Split subnodes have their own Gini index. We calculated the Gini index before node *m* splitting, denoted as $V\,I\,M_{i\,m}^{\left(G\,i\,n\,i\right)}$, and Gini index of two subnodes after splitting denoted as *GI*_*l*_ and *GI*_*r*_, respectively. The bigger the $V\,I\,M_{i\,m}^{\left(G\,i\,n\,i\right)}$, the more important the *i*-th gene.

(3) $$V\,I\,M_{t\,i}^{\left(G\,i\,n\,i\right)}=\sum_{m\in M}V\,I\,M_{i\,m}^{\left(G\,i\,n\,i\right)}$$

*T* was used as a set of trees, and *t* denotes the *t*-th tree. Equation 3 shows the importance of the *i*-th gene in the *t*-th tree. Thereafter, we calculated the importance of the *i*th gene in all trees, and the sum was represented as Eq. 4 depicts:

(4) $$V\,I\,M_{i}^{\left(G\,i\,n\,i\right)}=\sum_{t=1}^{T}V\,I\,M_{t\,i}^{\left(G\,i\,n\,i\right)}$$

Finally, importance scores of each feature in all trees were averaged by weight. The importance of each gene sorted according to their averaged importance score. We selected the top *n* genes by importance score, where *n* was a flexible value set to obtain the best classification performance.

### Logistic Regression Classifier

We used the logistic regression algorithm to construct a classifier (Zhang et al., 2014; Pranoto et al., 2015). Logistic regression uses the sigmoid function to represent the probability of a sample being labeled as a certain category, and prediction of tissue origin can be explained as a one-to-many classification problem. In this process, one type of cancer was considered positive, and other types were considered negative. Thereafter, the probability of the sample was predicted as one cancer type and other cancer types, respectively. After a series of similar procedures, we obtained the probability of a sample being predicted as each cancer. The prediction function was calculated by Eq. 5:

(5) $$h_{\theta }\,\left(x\right)=\frac{1}{1+e^{-\theta^{T}\,x}}$$

where *h*_θ_(*x*) denotes the probability of a sample being predicted as one cancer type (positive), or other cancer types, (negative). θ^*T*^ is a matrix of parameters used to determine the best model. θ is computed by the negative log-likelihood loss function. The loss function was calculated by Eq. 6:

(6) $$\text{J}\left(\text{θ}\right)=-\frac{1}{m}\left[\sum_{i=1}^{m}y^{\left(i\right)}\log h_{\text{θ}}\left(x^{i}\right)+\left(1-y^{i}\right)\text{log}\left(1-h_{\text{θ}}\left(x^{i}\right)\right)\right]+\frac{\text{λ}}{2m}\sum_{j=1}^{n}{\text{θ}}_{j}^{2}$$

where *logh*_θ_(*x*^(*i*)^) and *log*⁡(1−*h*_θ_(*x*^(*i*)^)) represent the log loss when a sample is labeled positive and negative, respectively. *m* represents the number of samples, and *n* denotes the number of features. And L1 regularization term was also used. The best θ was determined by minimizing the loss function based on gradient descent.

### Evaluation Metric

We used accuracy, precision, recall, and F1 score as the metric for performance evaluation. True positive (TP) and false positive (FP) represent samples whose true label are positive and negative, respectively, were predicted as positive, whereas true negative (TN) and false negative (FN) represent samples, whose true label was negative and positive, respectively. These were predicted as negative. Accuracy was used to measure the overall performance and was calculated by Eq. 7. Precision demonstrates the ability of classifier to distinguish positive and negative samples and was calculated by Eq. 8. Recall represents the ability of the classifier to recognize all positive samples and was calculated by Eq. 9. F1 score was the harmonic average value of precision and recall and is calculated by Eq. 10. Because there is class imbalance in sample distribution in this study, ROC (receiver operating characteristic) curve and AUC (area under the curve) were also used to evaluate classification performance.

(7) $$\text{Accuracy}=\,\frac{\text{TP}+\text{TN}}{\text{TP}+\text{TN}+\text{FP}+\text{FN}}$$

(8) $$\text{Precision}=\,\frac{\text{TP}}{\text{TP}+\text{FP}}$$

(9) $$\text{Recall}=\,\frac{\text{TP}}{\text{TP}+\text{FN}}$$

(10) $$\text{F}\,1\,\mathrm{score}=\,\frac{2\,\text{TP}}{2\,\text{TP}+\text{FP}+\text{FN}}$$

### Functional Annotation

We utilized the Gene Ontology enrichment analysis database (Ye et al., 2006; Waardenberg et al., 2016) to annotate the function of the gene used in the model, shown in Figure 3. The R package gogadget and clusterProfiler (Nota, 2016; Yu et al., 2012) were used for gene visualization and clustering.

## Results

### Workflow

**FIGURE 1:**
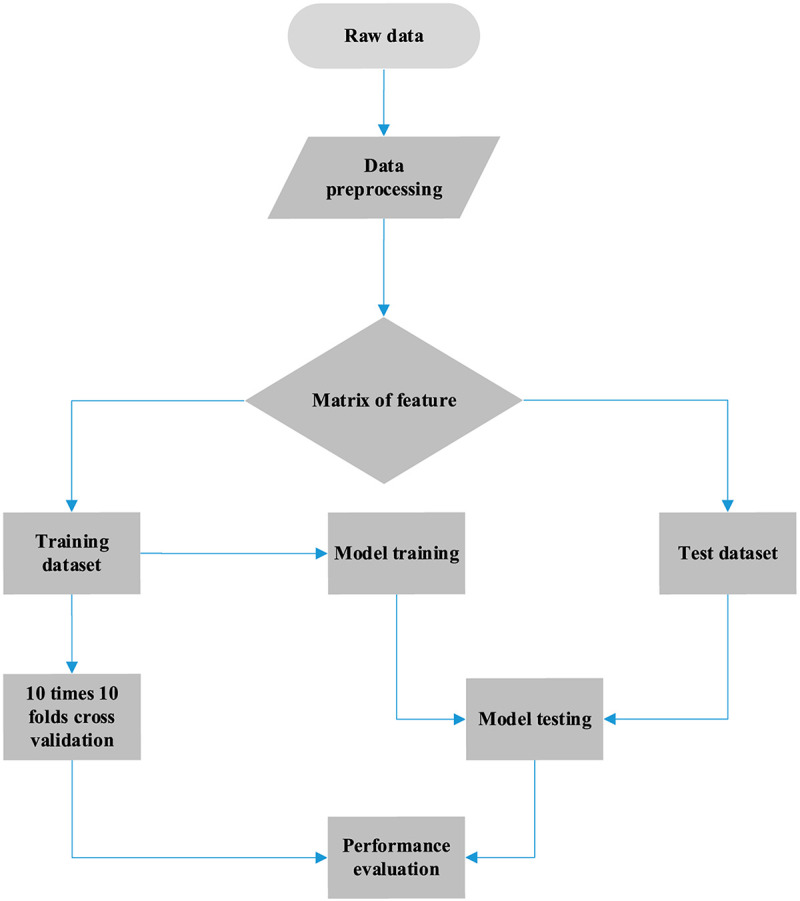
Workflow of cancer tissue origin identification using somatic mutation data.

The complete process for predicting the primary site of a tumor is shown in Figure 1, which can be divided into three parts. First, we obtained the somatic mutation data from the ICGC database and carried out data preprocessing such as filled null value and filtered invalid data. A matrix of features was generated for follow-up handling. Thereafter, we built a gene selection model using the random forest algorithm. Genes were selected with 10-time cross-validation. Finally, we constructed the classifier by utilizing the logistic regression algorithm, and the final matrix feature was fed into the classifier. The results were obtained with 10-time 10-fold cross-validation, and model performance was analyzed by the evaluation metric.

### Data

We obtained the somatic mutation data from ICGC version 28 database for gene selection and tumor classification. Allelic mutations in somatic mutation data can be A/G, C/T, C/A, and so on. Because of limited information and tools, we treated all allele mutations as mutations and counted the number of mutations. And we counted the number of mutations of each sample. The sample distribution of each cancer is shown in Table 1. A total of 3,219 primary samples and 155 metastatic samples were used to model training and included 13 types of cancer.

### Genes Used to Infer Cancer Tissue of Origin

**FIGURE 2:**
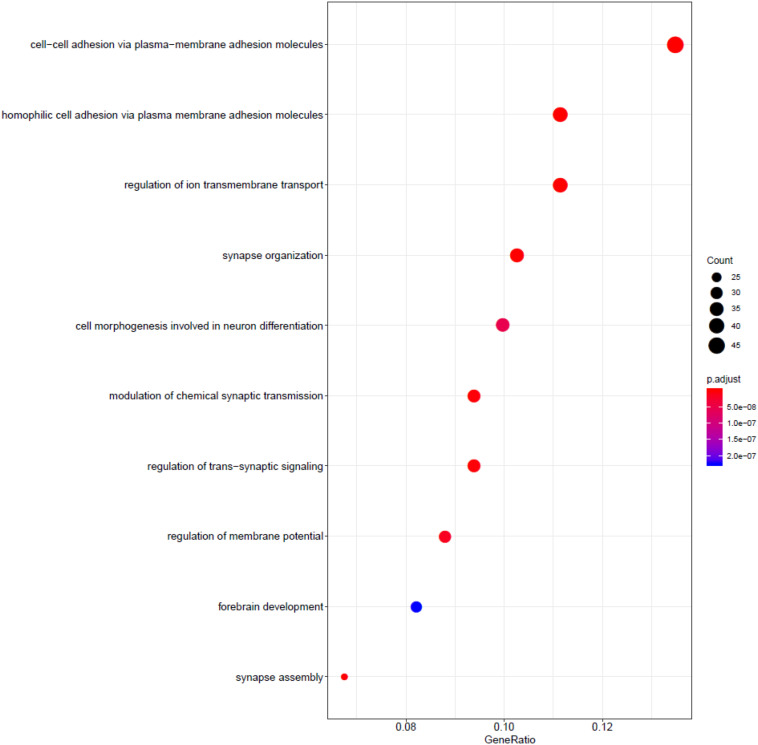
Functional annotation of the top 500 genes.

The role of relative genes was discussed in context of molecular function, biological processes, and cellular components. Figure 2 shows functional annotation of the top 500 genes selected using the random forest algorithm. Genes were found to enrich cell–cell adhesion, regulation of ion transmembrane transport, modulation of chemical synaptic transmission, forebrain development, and so on. Among these, gene enrichment evidently concentrated on the recognition and adhesion between cells and neurotransmitter conduction. Abnormal proteins that resulted from gene mutations can cause abnormal cell adhesion or differentiation, as well as abnormal neurotransmitter conduction or abnormal neural cell differentiation. Meanwhile, gastric cancer and brain lower-grade glioma account for a high proportion in all samples. Jiang et al. (2004) research the frequency and nature of mutations of the *CDH1* gene in gastric cancer, and proved that the mutation accounts for gastric cancer. The *APC* gene has been found to play an important role in the pathogenesis of soft tissue tumors (Kuhnen et al., 2000). Birnbaum et al. (2012) explored the role of the *APC* gene in colorectal cancer, by investigating 183 cases, and found point mutations in 73% of these cases. Mutation of the *IDH1* gene leads to a reduction in cell survival and proliferation, as well as further invasion of human gliomas by malignant tumor cells (Cui et al., 2016). Mutation of the *IDH1* gene has been proved to be the driving oncogenic factor of and has an impact on most brain lower-grade gliomas of different genetic pathways (Ohno et al., 2013; Pieper et al., 2014; Ohka et al., 2017).

**FIGURE 3:**
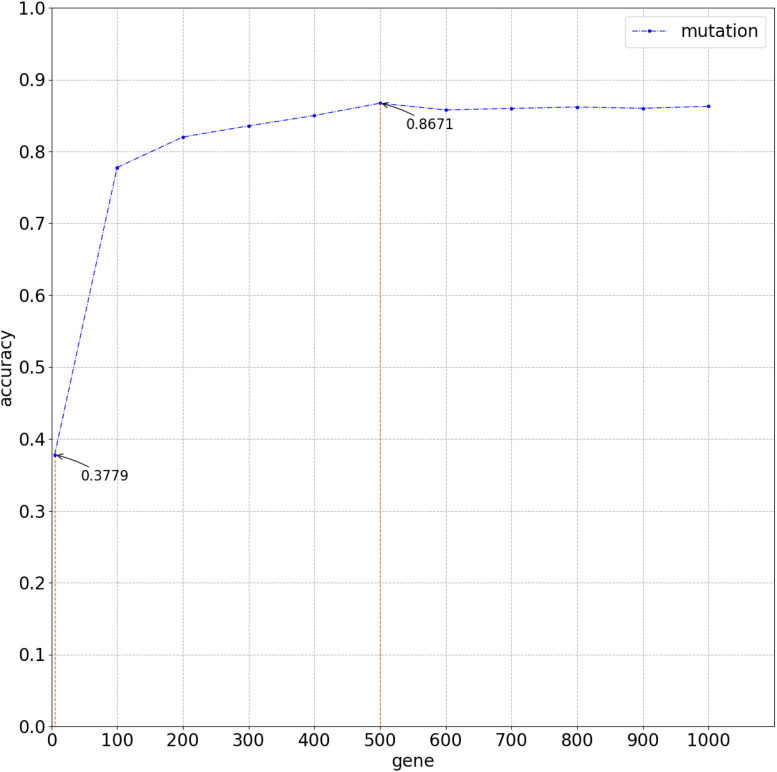
Overall average accuracy using logistic regression classifier with 10-time 10-fold cross-validation.

According to research carried out on patients with liver cancer from China and southern Africa, a mutational hotspot at codon 249 of the p53 tumor suppressor gene has been identified (Hsu et al., 1993), and HBV and aflatoxin B1 (AFB1) are known synergistic risk factors. Zheng et al. (2005) explored the role of mutation of the DNA polymeraseβ (polβ) gene in human nasopharyngeal cancer and its relationship with EBV. Zhao (2001) carried out investigation on the mutation of the *ras* gene and what role they played in HP infection. They determined the infection of HP through serological examination. The results showed that 28 of 43 cases existed with mutations in codon 12 and a mutation rate of 65.12% (Zhao, 2001). Supplementary Figure 1 also shows the relationship between gene mutations and cancers. Therefore, we concluded that viral infections could lead to gene mutations and result in cancer. In this study, somatic mutation data were utilized to identify the primary site of a tumor based on machine learning methods, which can contribute to the further diagnosis and treatment of cancer.

### Performance Evaluation

**FIGURE 4:**
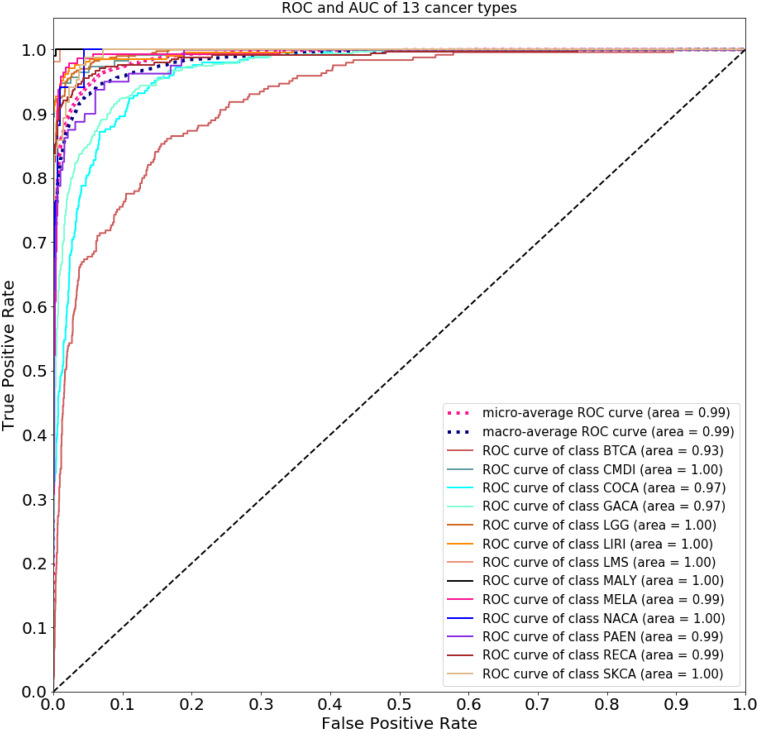
Receiver operating characteristic curve and AUC of 13 types of cancer.

Figure 2 compares the accuracy with a different number of genes used in the classifier. Because of gene sequencing and mutation detection being costly and time consuming, we selected 100 and 1,000 as the minimum and maximum number of genes, respectively. And we carried out a large number of experiments, with 100 genes selected as the interval. The highest accuracy was obtained when using the top 500 genes. These results are shown in Figure 3 with 10-time 10-fold cross-validation. The average accuracy is 86.71%, and precision, recall, and F1 score are presented in Table 2. The ROC curve and AUC of 13 types of cancer are shown in Figure 4. Most curves are close to 100%, and the area of each cancer is very close to 1 except BTCA (biliary tract cancer). The micro-average and macro-average are 0.99, which show the prediction value of each dimension and the average of all areas. Combining the metrics of prediction accuracy, ROC, AUC, and so on, our model had the worst overall prediction performance at biliary tract cancer and the best overall prediction performance at malignant lymphoma. Liver cancer, nasopharyngeal cancer, and gastric cancer are caused by HBV, HCV, EBV, and HP, respectively. The performance of our model on nasopharyngeal cancer was comparatively poor. In general, our model can obtain considerable prediction performance with the use of mutation data, which is great help in identification of the primary site of a tumor, follow-up diagnosis, and treatment.

**TABLE 2:** Performance metric of training dataset using top 500 genes.

Cancer	Precision	Recall	F1 score	Support	Specificity
BTCA	0.6288	0.6331	0.6308	245.0000	0.9626
CMDI	0.9789	0.8921	0.9335	114.0000	0.9991
COCA	0.6479	0.7700	0.7036	250.0000	0.9573
GACA	0.8556	0.8265	0.8408	570.0000	0.9627
LGG	0.9315	0.9178	0.9246	400.0000	0.9883
LIRI	0.9390	0.9362	0.9376	207.0000	0.9949
LMS	0.9981	0.9796	0.9888	54.0000	1.0000
MALY	0.9944	0.9893	0.9918	196.0000	0.9996
MELA	0.8851	0.9147	0.8996	143.0000	0.9934
NACA	0.9018	0.6118	0.7275	17.0000	0.9996
PAEN	0.7150	0.7738	0.7431	80.0000	0.9906
RECA	0.9294	0.9077	0.9184	339.0000	0.9901
SKCA	0.9251	0.8259	0.8726	85.0000	0.9978
Average	0.8552	0.8445	0.8548	2, 700.0000	0.9883
Accuracy	0.8671	NA	NA	NA	NA

In this study, the metastatic samples were used as test dataset. We carried out experiments by using 500 chosen genes with use of the model trained by training dataset. An average classification accuracy is 86.39%, as shown in Table 3. Although the model performed poorly on Pancreatic endocrine neoplasms (PAEN), the overall classification accuracy is satisfying. In this condition, we considered that little error on classification is tolerable.

**TABLE 3:** Performance metric of test dataset using top 500 genes.

Cancer	Precision	Recall	F1 score	Support	Specificity
BTCA	0.6429	0.6000	0.6207	15.0000	0.9675
CMDI	1.0000	1.0000	1.0000	5.0000	1.0000
COCA	0.7059	0.7500	0.7273	16.0000	0.9673
GACA	0.8148	0.7097	0.7586	31.0000	0.9638
LGG	0.9412	1.0000	0.9697	32.0000	0.9854
LIRI	0.9412	0.8889	0.9143	18.0000	0.9934
LMS	1.0000	1.0000	1.0000	2.0000	1.0000
MALY	1.0000	1.0000	1.0000	9.0000	1.0000
MELA	1.0000	0.8889	0.9412	9.0000	1.0000
NACA	1.0000	1.0000	1.0000	2.0000	1.0000
PAEN	0.3333	1.0000	0.5000	1.0000	0.9881
RECA	0.9583	0.9583	0.9583	24.0000	0.9931
SKCA	0.7143	1.0000	0.8333	5.0000	0.9878
Average	0.8501	0.9074	0.8633	169.0000	0.9890
Accuracy	0.8639	NA	NA	NA	NA

**FIGURE 5:**
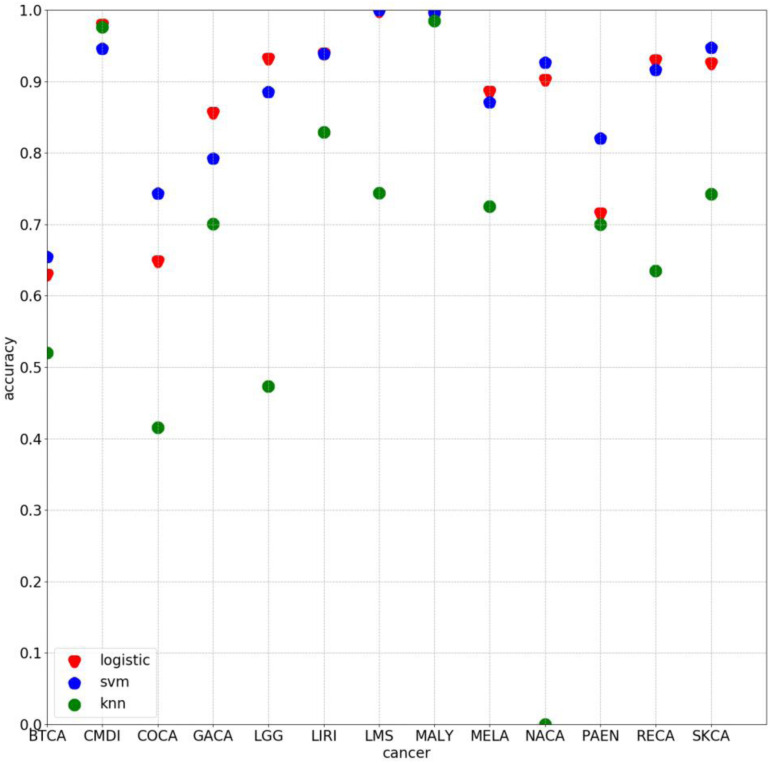
Classification accuracy on each cancer by using 500 chosen genes based on logistic, svm, and knn, respectively.

Some experiments were also conducted by using other algorithm with 500 selected genes. The average classification accuracy values of using k-nearest neighbor (knn) and support vector machine (svm) are 62.66 and 85.27%, respectively, lower than 86.71% obtained by using the method proposed in this study. As Figure 5 clearly shows, the classification accuracy on each cancer of using logistic algorithm was significantly higher than using knn. The overall performance of logistic is also better than svm. Therefore, the method proposed in this study can provide better prediction performance.

### Mean Value of Number of Somatic Mutations on Each Cancer

We mapped the number of somatic mutations in each cancer, as shown in Supplementary Figure 1. Columns represent cancers, and rows represent genes. The number of mutations is colored on a logarithmic scale. Also, we used the color bar to show difference in values. The color of rectangles in the heat map represents the relative log number of mutations per gene in each cancer type. Cancers distributed in clusters along the vertical axis had similar values in the number of mutations. Genes also cluster on the horizontal axis, based on the association between cancers.

## Discussion

Viruses have been proven an important cause of cancer (Tsai et al., 1996; Lin et al., 2013; Geng and Zhang, 2017). Achieving effective identification of the primary site of a tumor caused by viruses or other factors plays a vital role in the follow-up diagnosis and treatment. Existing research shows that molecular profiling can be used to predict the primary site of a tumor. In this study, somatic mutation data were used to determine cancer tissue origin. Samples of 13 types of cancer were used with 3,374 samples used for feature extraction. The selected top 500 genes with mutation data were selected based on the feature importance score and was trained in the proposed classifier with 10-time 10-fold cross-validation. An average accuracy of 86.71% was obtained with use of machine learning algorithms, random forest algorithm, and logistic regression, utilized for gene selection and cancer classification, respectively.

Our model can achieve considerable performance in prediction of the primary site of common cancers caused by a virus or other factors. However, prediction performances on biliary tract cancer and nasopharyngeal carcinoma are discouraging. According to the sample distribution in Table 1, poor performance on nasopharyngeal carcinoma may be attributed to the small quantity of samples tested for this carcinoma. The reason for poor classification of the biliary tract cancer requires further research because of a lack of evidence. Therefore, we infer that there are shortcomings in using mutation data alone to identify the primary site of some cancers, but our model can obtain considerable overall performance. This positively affects the follow-up diagnosis and treatment.

## Conclusion

As a large number of patients have CUP, tracing the primary site of a tumor has been a long-term challenge. Molecular profiling of tissue-specific genes is available from public database or medical institutions. We conducted experiments using somatic mutation data based on machine learning algorithms. Results showed that the proposed method is beneficial to the diagnosis and treatment of patients with unknown primary sites. However, the model does not perform well on all cancers. This motivates for further research on the identification of tissue origin of more common cancers. And research on performance of combination of somatic mutation data and other molecular profiling will be considered in our future work. Currently, the proposed method can achieve considerable performance and will help in the progress of the follow-up study.

## Data Availability Statement

Publicly available datasets were analyzed in this study. This data can be found here: https://dcc.icgc.org/releases/release_28/.

## Author Contributions

LZ and HZ designed the project. XL and LL analyzed the data, carried out the experiments, and wrote the manuscript. LP, BW, JL, QL, GT, XZ, and YS modified and reviewed the manuscript. All authors contributed to the article and approved the submitted version.

## Conflict of Interest

JL, BW, QL, and GT are employed by Genesis Beijing Co., Ltd. The remaining authors declare that the research was conducted in the absence of any commercial or financial relationships that could be construed as a potential conflict of interest.

## Footnotes

1 https://dcc.icgc.org/releases/release_28/
